# Supplementary figures and images for: Large-scale phenotyping and comparative genomics reveal genetic features of Listeria persistence in epithelial cells
Source: PLoS Pathog. 2026 Apr 15;22(4):e1013323. doi: 10.1371/journal.ppat.1013323 (PMC13082608; doi:10.1371/journal.ppat.1013323)

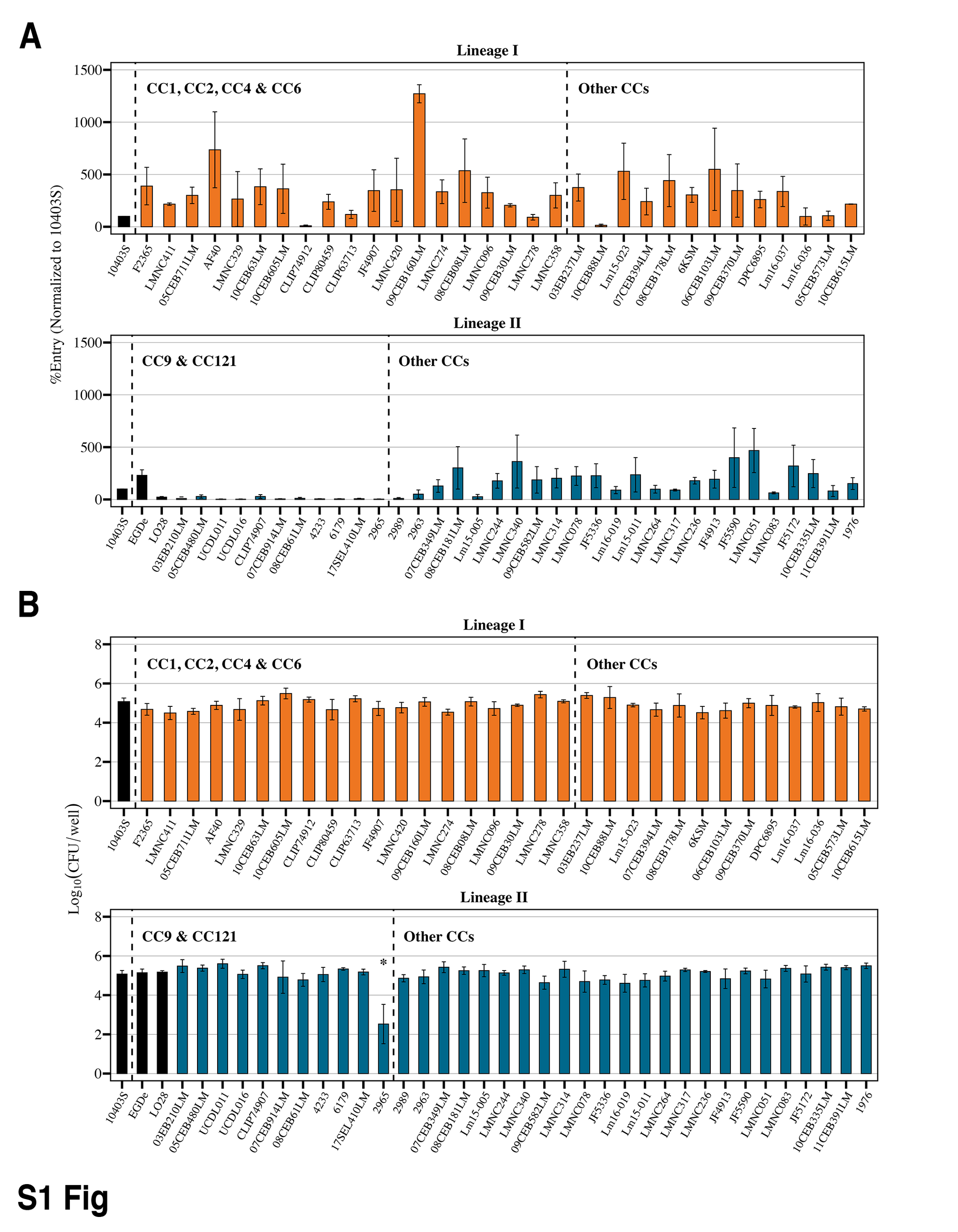

Supplement: S1 Fig — Entry capacity at 2h p.i. (A) and intracellular multiplication capacity at 72h p.i. (B) were analyzed according to strains, clonal complexes and lineages. Laboratory strains 10403S, EGDe and LO28 are shown in black. The 2965 variant (V1), which exhibits a significant reduction in intracellular bacterial load, is indicated with an asterisk. Results represent the mean of three independent experiments. Statistical significance was determined using a two-Sample t-test comparing each strain to the reference strain10403S, with Holm’s adjustment for p-value (*: p ≤ 0.05). (TIF) [file ppat.1013323.s001.tif]

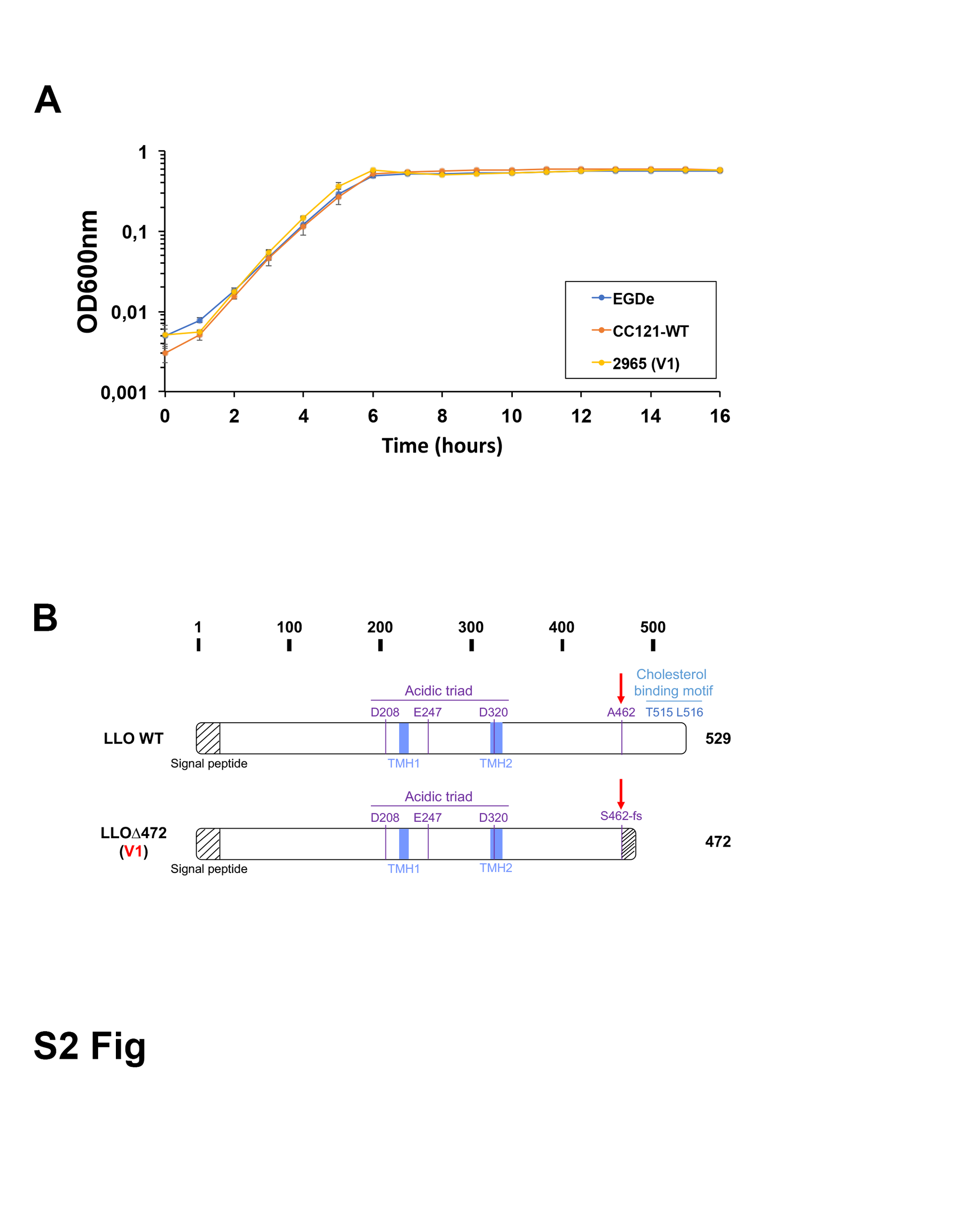

Supplement: S2 Fig — A. Growth curve of the 2965 variant (V1) in BHI medium, compared to the reference strains EGDe and CC121-WT (17SEL410LM). B. Schematic representation of listeriolysin O (LLO) from the reference strain EGDe (top) and the V1 variant (bottom). The signal peptide, pH sensor (acidic triad D208, E247, D320), and cholesterol-binding motif are indicated, with the two transmembrane β-hairpins (TMH) highlighted in blue. In V1, a mutation at position 462 (red arrow) induces a frameshift (fs), resulting to the loss of the 67 C-terminal amino acids, which are essential for membrane binding and proper LLO function. (TIF) [file ppat.1013323.s002.tif]

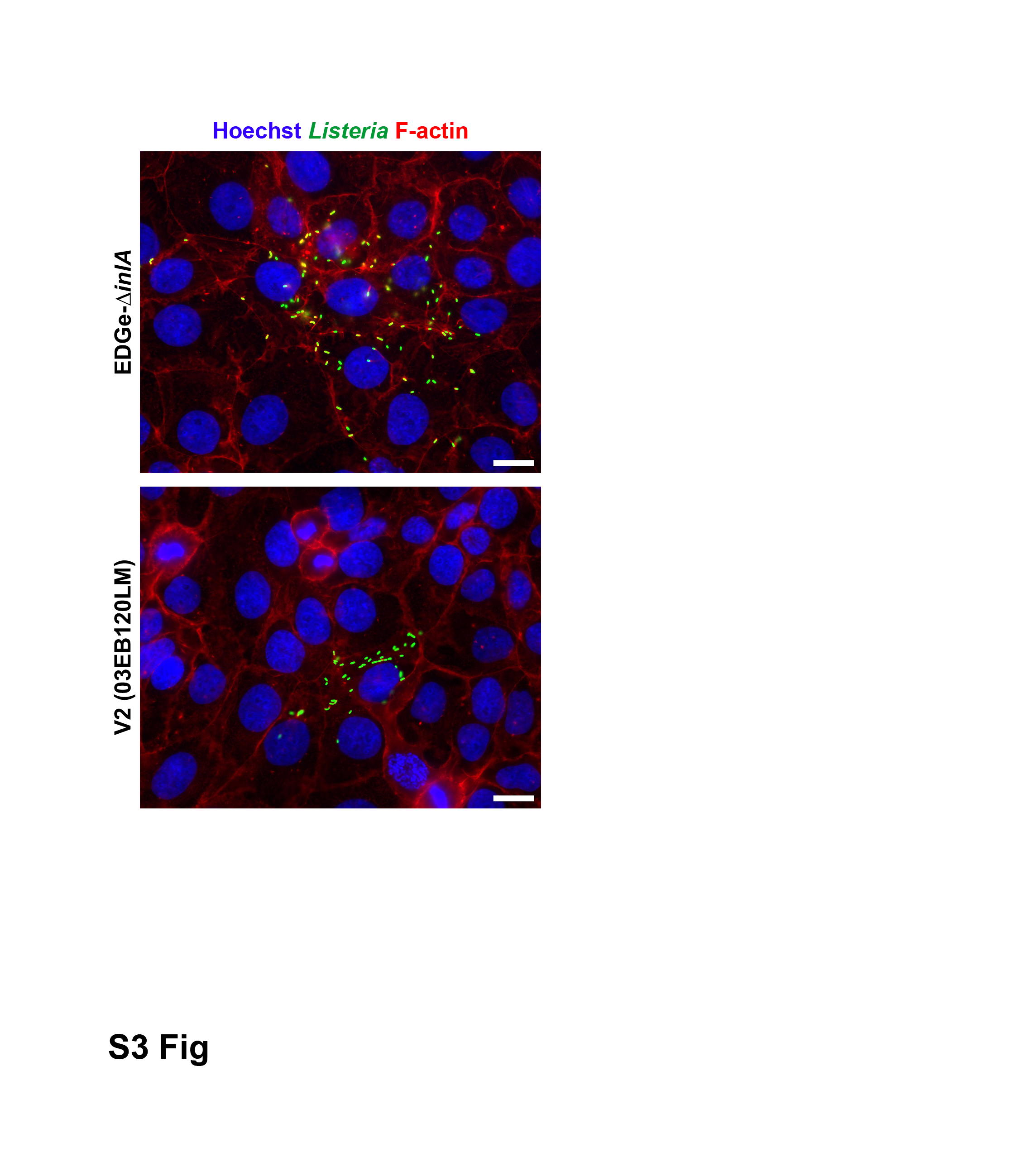

Supplement: S3 Fig — High magnification micrographs of JEG-3 cells infected for 6 h with EGDe-∆inlA (top panel) and V2 (03EB120LM, bottom panel). Images show overlays of Hoechst (blue), Lm (green) and F-actin (red) signals. Bars: 20 μm. (TIF) [file ppat.1013323.s003.tif]

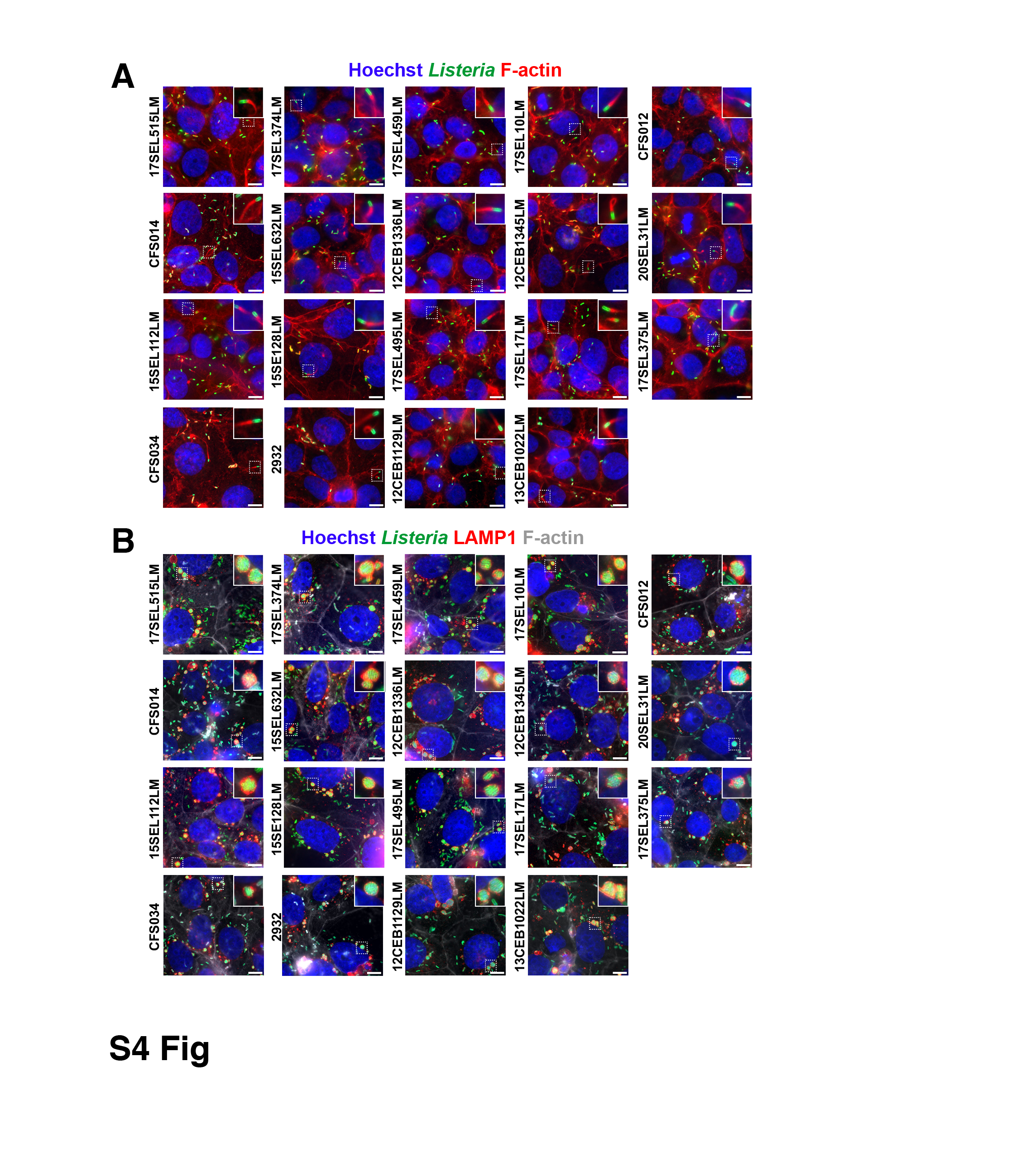

Supplement: S4 Fig — ) and LisCVs formation (72 h p.i.). A. Representative micrographs of JEG-3 cells infected for 6 h with the indicated strains. Images show overlays of Hoechst (blue), Lm (green) and F -actin (red) signals. Bars: 10µm. Insets highlight high-magnification views of the boxed regions with bacteria forming actin comets. B. Micrographs of JEG-3 cells infected for 72 h with the indicated strain. Images show overlays of Hoechst (blue), Lm (green), LAMP1 (red) and F-actin (white) signals. Bars: 10µm. Insets show high-magnification views of the boxed regions with representative LisCVs. (TIF) [file ppat.1013323.s004.tif]

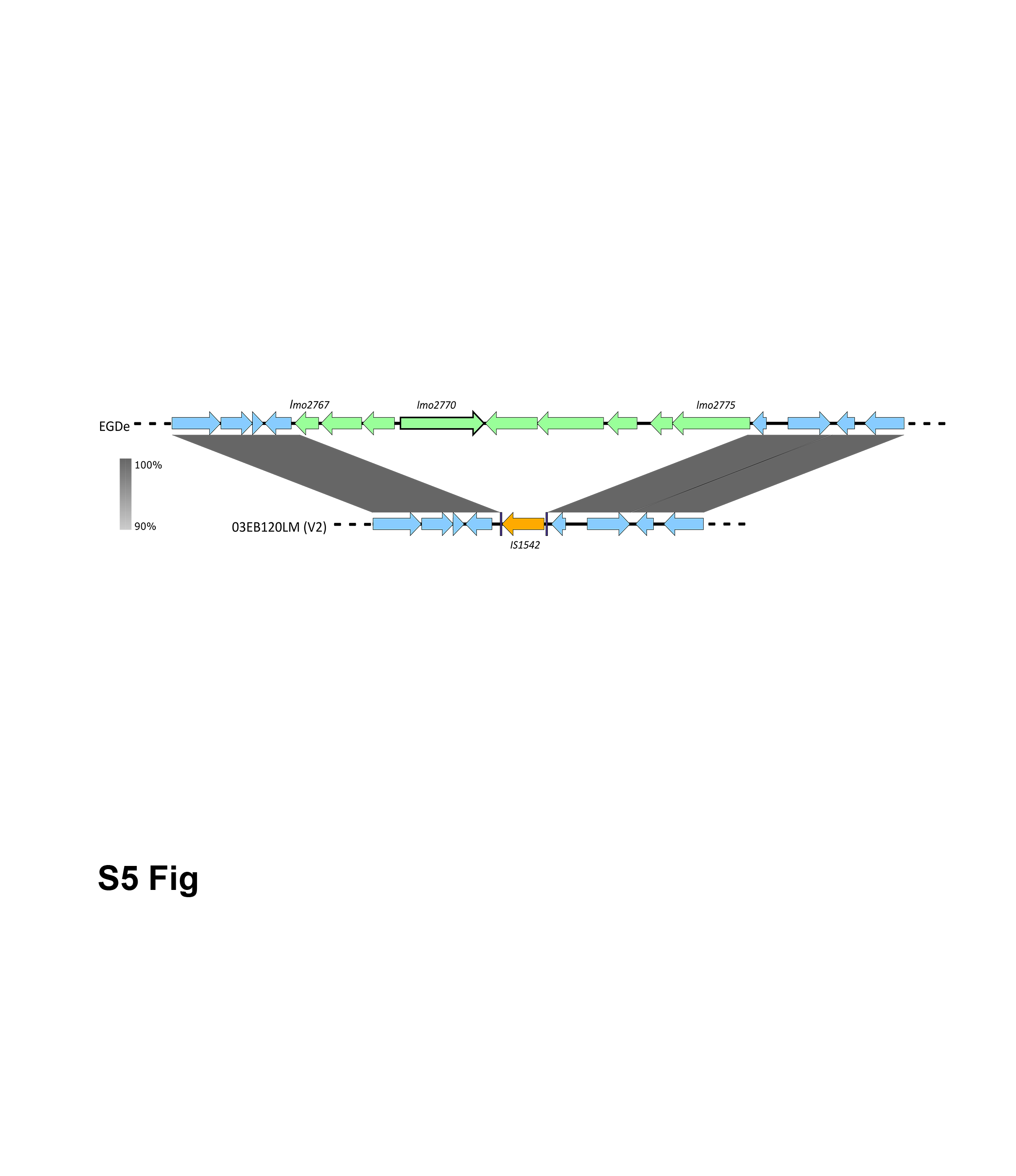

Supplement: S5 Fig — The 9 genes present in EGDe but absent in the V2 variant are shown in green, with the gshF gene, encoding for the glutathione synthase, highlighted in bold. The IS1542, that substitutes the gshF region, is depicted in orange. The two inverted repeats flanking the transposase are represented by purple rectangles. Genome comparisons were visualized using Easyfig, version 2.1 [73]. (TIF) [file ppat.1013323.s005.tif]

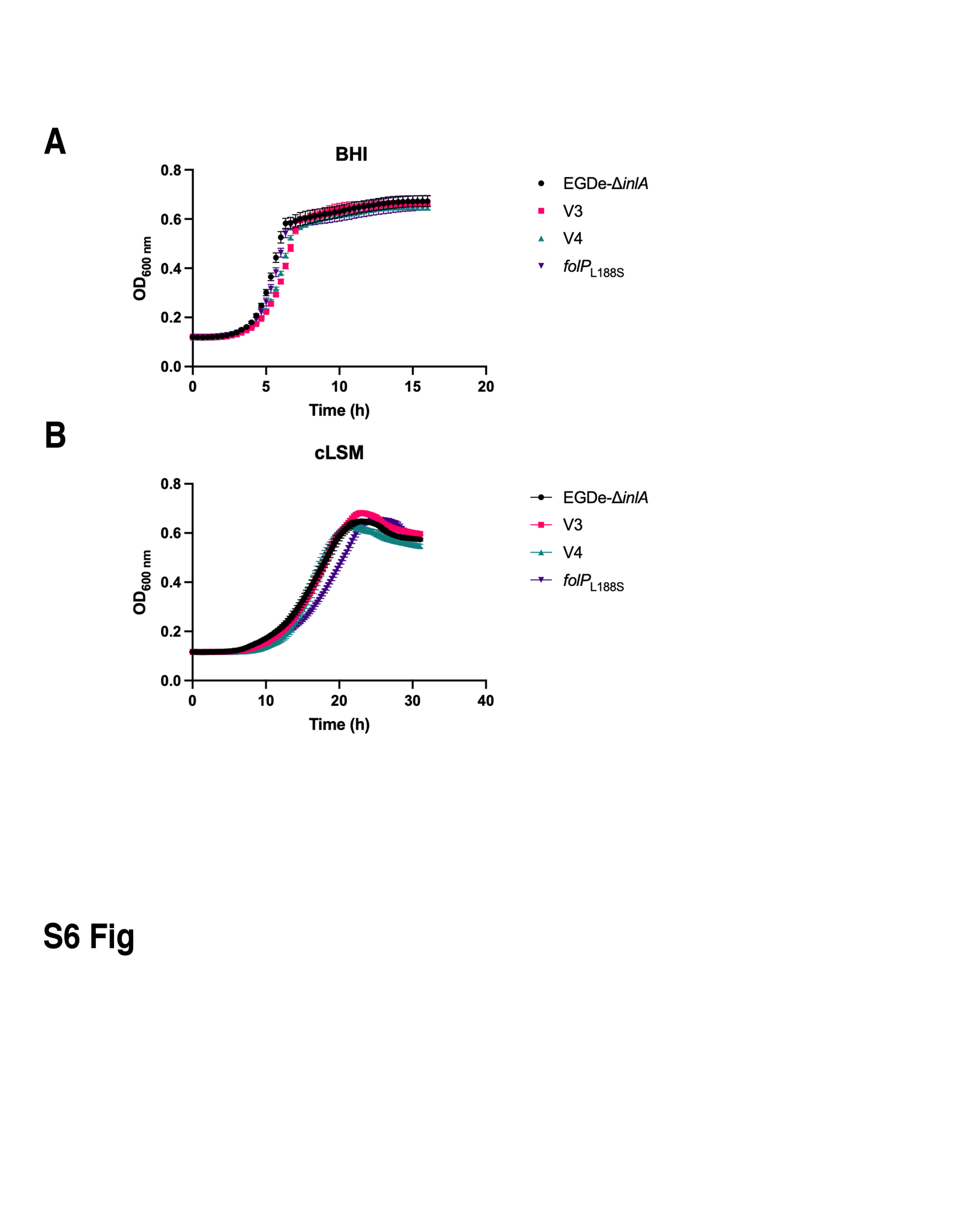

Supplement: S6 Fig — (TIF) [file ppat.1013323.s006.tif]
