# Supplementary material for: Large-scale phenotyping and comparative genomics reveal genetic features of Listeria persistence in epithelial cells
Source: PLoS Pathog. 2026 Apr 15;22(4):e1013323. doi: 10.1371/journal.ppat.1013323 (PMC13082608; doi:10.1371/journal.ppat.1013323)
Supplement: Attachment — Submitted filename: Response to Reviewers.pdf [file ppat.1013323.s014.pdf]

Part I - Summary

Please use this section to discuss strengths/weaknesses of study, novelty/significance, general execution and scholarship.

Reviewer #1: In this study, the authors investigate the ability of Listeria monocytogenes (Lm) strains from diverse clonal complexes (CCs) to form LisCVs, LAMP1-positive vacuoles associated with Lm persistence in epithelial cells. Using 105 isolates of different orgins, they demonstrate that LisCV formation is broadly conserved across CCs, regardless of their source. Among these, three isolates (V2, V3, and V4—all CC9 with inlA PMSC mutations) exhibited altered LisCV phenotypes. The authors show that ectopic expression of gshF in V2 restores ActA expression and increases LisCV size. They also identify a folP L188S mutation in V3 and V4 that reduces Lm proliferation, cell-to-cell spread, and LisCV size, while paradoxically increasing ActA surface expression in EGDe. These findings suggest that ActA regulation plays a key role in Lm persistence, especially among hypo-virulent CC9 strains, while the role of actA in LisCV was already demonstrated previously (Kortebi et al., 2017). As highlighted in the abstract and author summary, the study's central novel finding is the identification of folP as a regulator of ActA expression and function, likely mediated through impaired folate biosynthesis, given that EGDe expressing folP L188S fails to synthesize folate at wild-type levels. However, the underlying mechanism remains unclear.

Reviewer #2: Listeria is now appreciated to persist within host tissue, in part, by growing within vacuolar compartments in host cells. In this manuscript, the authors use a phenotyping and compartative genomics approach to identify genetic features that impact Listeria persistence in epithelial cells. They identify new variants in hly, gshF and folP genes that impact Listeria persistence. Mechanistically they find that ActA expression and function are critical for generation of the persistence niche. The manuscript addresses an important and understudied aspect of Listeria infections and, through the use of many clinical isolates, supports the emerging view that persistence in host cells is a critical virulence trait. The paper is well written and the provides a roadmap for future studies of its kind. I include my comments for the authors consideration.

Part II – Major Issues: Key Experiments Required for Acceptance

Reviewer #1: While elucidating how folP L188S affects ActA function may be challenging, the authors could strengthen their claim by addressing whether ActA is required for LisCV modulation in the isolates (V2, V3 and V4). This could be tested using EGDe folP L188S combined with inlA and actA deletions. A similar approach should be applied to gshF complementation in V2: introducing actA deletion in V2 or in V2 expressing gshF would clarify whether ActA mediates the observed LisCV phenotypes.

We have addressed the reviewer's comment by performing the suggested experiments.

To directly test whether the vacuolar phenotype of the *folP L188S* mutant results from ActA deregulation, we deleted *actA* in the *folP L188S* background and analysed LisCV biogenesis at 72 h post-infection. As expected, *actA* deletion was epistatic: the Δ*actA*-*folP L188S* strain fully phenocopied the Δ*actA* mutant. We have included these data in a new panel (see new Fig.5J).

To further clarify the contribution of ActA in the LisCV phenotype, we introduced the *actA* deletion into both the V2 and V2 strain expressing *gshF*. The V2-Δ*actA* strain displayed a vacuolar phenotype comparable to that of the V2 strain in terms of of LisCV size. Moreover, expression of *gshF* in the V2 strain was no longer able to restore LisCV size in the absence of ActA . These new data are shown in Figs 3H–I.

All in vitro experiments were performed using JEG3 trophoblastic cells, which are permissive to both InlA and InlB, even when using inlA-deficient strains. To extend the physiological relevance of LisCVs, it would be valuable to demonstrate their presence in vivo, such as in the placenta of wild-type mice, where InlA is dispensable, or in the liver, where LisCVs were previously observed in hepatocytes (Kortebi et al., 2017). In vivo evidence of LisCV formation and modulation would substantially enhance the study's impact by underscoring its significance in Lm persistence and pathogenesis.

We agree with the Reviewer that demonstrating the presence of LisCVs *in vivo* is an important step. However, the experimental confirmation that LisCVs exist *in vivo* is a laborious and time-consuming work that cannot be realistically achieved within the timeframe of a revision. Demonstrating the presence of persistent intravacuolar *Lm* would require the development of an infection model that has not yet been used in the *Listeria* field. By definition, persistent *Lm* are carried asymptomatically, which would need either antibiotic treatment of infected animals or infection with sublethal bacterial doses. Furthermore, intravacuolar dormant *Lm* are likely to be present in very low numbers, making their histological detection particularly challenging. It is also possible that the mouse model may not be suitable for studying persistent *Lm* in epithelial tissues, and alternative animal models or human tissues might be required to validate our observations. We are actively working on these aspects, but establishing and optimizing such experimental approaches will require significant time and effort.

It is worth noting that the first report of the most iconic intracellular phenotype of *Lm* (bacteria associated with actin filaments– Tilney and Portnoy *J Cell Biol*, 1989 1989) was a very descriptive yet seminal publication. Furthermore, although comet tails are extremely well documented using tissue culture cells, bacteria associated with actin have very rarely been observed within mammalian hosts. The only convincing published electron micrographs from *in vivo* specimens date back to a 1972 study (Racz, et al *Lab Investig*, 1972). Two other studies suggested the presence of actin-associated bacteria within mammalian tissues, but did not clearly demonstrate it (Gaillard et al *J Exp Med*, 1996; Henke et al *Infect Immun,* 2015). Together, these examples illustrate the difficulty of studying intracellular *Lm* phenotypes *in vivo*.

In our study, using a combination of complementary approaches and diverse *Lm* isolates we established, for the first time, that bacterial persistence inside LisCVs is not restricted to the laboratory strains (EGDe and 10403s) and identified key molecular players involved in this poorly characterized phase. We believe our findings provide a solid foundation for future *in vivo* studies that are nevertheless beyond the scope of the present study.

Impaired folate metabolism and ActA deregulation are not necessary on the same stream. Is it possible to add back FolP downstream metabolites, such as folic acid shown in Fig 5J, to address the role of folate metabolism on the ActA deregulation?

To investigate whether impaired folate metabolism directly contributes to ActA deregulation, we first attempted a metabolic complementation approach (see Fig. below). Specifically, we supplemented infected Jeg-3 cells with DHF (7,8-dihydrofolate), a downstream metabolite of FolP, during cellular infection. Unexpectedly, DHF supplementation resulted in a deleterious effect on bacterial replication in both the wild-type and the *folP*L188S

strains. At present, we do not have a clear explanation for this effect, which prevented us from using this approach to directly assess ActA regulation.

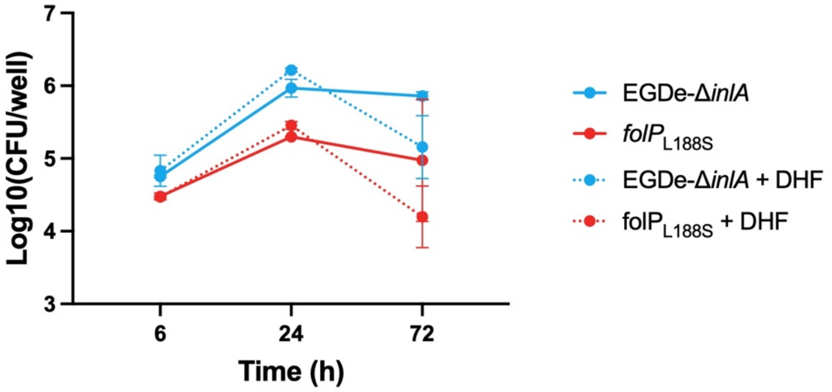

We therefore adopted a genetic complementation strategy. We complemented with *folP*wt the EGDe-Δ*inlA* *folP*L188S strain. Complementation restored LisCV size, as well as infection foci size and surface-associated ActA levels, along with intracellular folate concentrations, to levels comparable to those of the EGDe-Δ*inlA* reference strain. These results support a functional link between folate metabolism and ActA regulation. They are presented in the revised manuscript in Figs 5B, 5E, 5G and 5K.

Reviewer #2: -Fig1D. Are these bacteria in LisCV or eSLAPs? Is there a marker the authors can use to test this? This question does not take away my enthusiasm for these studies of Listeria persistence in vacuoles. But it is important the authors use the two terms accurately.

We are glad that the Reviewer is enthusiastic about our work and genuinely appreciate that.

We are convinced that the vacuoles observed in Fig. 1D are not eSLAPs, for the following reasons.

First, eSLAPs are transient internalization vacuoles that persist only for a few hours before rupturing and releasing *Listeria* into the cytosol (Peron-Cane *et al PloS Pathog*, 2020). In contrast, the LAMP1-positive vacuoles shown in Fig. 1D are present 72 h post-infection, making highly unlikely that they represent internalization vacuoles, which are not known to remain stable for several days.

Second, eSLAPs represent only a very small proportion (~4%) of all entry vacuoles, as quantified by live-imaging experiments. In our experiments, conducted at a very low MOI (1 bacterium per 100 cells), it is practically impossible for these structures to be present in every infected cell. Notably, vacuoles are detected in virtually all cells and contain more than 50% of the total intracellular *Listeria*.

Third, we have previously shown that formation of LisVCs requires that bacteria first reach the cytosol (Kortebi *et al PloS Pathog*, 2017), whereas eSLAPs arise from entry events and do not require cytosolic access.

Finally, recent work demonstrated that eSLAPs do not form in JEG-3 cells (Petit et al, https://doi.org/10.1101/2025.09.02.673654)—the cell line we have used extensively throughout our study, including in the experiments shown in Fig. 1D.

Taken together, these observations strongly argue that the vacuoles we observe in Fig. 1D are not eSLAPs.

-There are many steps in the Listeria life cycle, and also in the formation of LisCVs. It would help the reader to include a schematic showing these steps early in the paper (e.g. Figure 1). This would help describing the different assays and where they fit into the paradigm of LisCV formation. It must also be borne in mind that changes to metabolism and virulence gene expression could impact any/all of the steps that lead up to LisCV formation.

We agree with the Reviewer that the intracellular lifestyle of *Listeria* is quite complex. However, we feel that including a schematic earlier in Fig.1 would extend beyond the focus of our study which is centered on LisCV biogenesis. Nevertheless, we have taken into account the reviewer's suggestion by adding few sentences that cites both the seminal works describing the vacuolar stages of *Listeria* and a recent review that discusses these stages in greater depth (Lines 81-90).

-Fig1D. Where are the images for V1? From Fig1E,F it seems there are very few bacteria, but where are they?

We apologize if this aspect of the manuscript was unclear or insufficiently supported by visual evidence. We have now added a new panel to Fig. 1D that shows individual V1 bacteria localized within LAMP1-positive internalization vacuoles. Of note, this phenotype is consistent with our previous findings using a *Δhly* mutant which is unable to escape entry vacuoles and therefore fails to replicate in the cytosol (Kortebi *et al PLoS Pathogens*, 2017). Similar to V1, the *Δhly* strain remains confined within perinuclear LAMP1-positive compartments that represent internalization vacuoles that have acquired lysosomal features.

-The authors should consider adding a schematic summarizing all the strains and mutations they identified linked to loss of Listeria persistence.

We have added a new table (Table 4) that compiles all variants identified in our study exhibiting either a hypovacuolar phenotype (less persistent) or a hypervacuolar phenotype (more persistent). For each variant, the corresponding genetic mutations are also indicated. We thank the reviewer for this suggestion.

Part III – Minor Issues: Editorial and Data Presentation Modifications

Please use this section for editorial suggestions as well as relatively minor modifications of existing data that would enhance clarity.

Reviewer #1: Fig. 1C. The change of intracellular bacterial load can be modulated by cell viability. Is there any information regarding the viability of the cells after 72 hours of infection? Did any strain kill the cells efficiently but show comparable intracellular bacterial load?

We thank the reviewer for raising this point. As mentioned in the manuscript (Lines 170-173), we did observe significant cytotoxicity for some lineage I strains, likely due to higher levels of LLO production or increased intracellular replication. For this reason, we clearly stated that CFU measurements at 72 hours do not fully capture the virulence potential of all isolates. However, our screening strategy specifically focused on identifying variants that displayed reduced intracellular CFU counts at 72 hours. The variants retained for further characterization (V1, V2, V3, and V4) did not exhibit detectable cytotoxic effects under our experimental conditions. Thus, their reduced intracellular loads cannot be attributed to host-cell death.

Caco2 is a human epithelial cell line used to identify inlA function. Are LisCVs observed in the cells?

We have performed this experiment and confirmed that Caco-2 cells are competent in LisCV formation. A representative image is provided below.

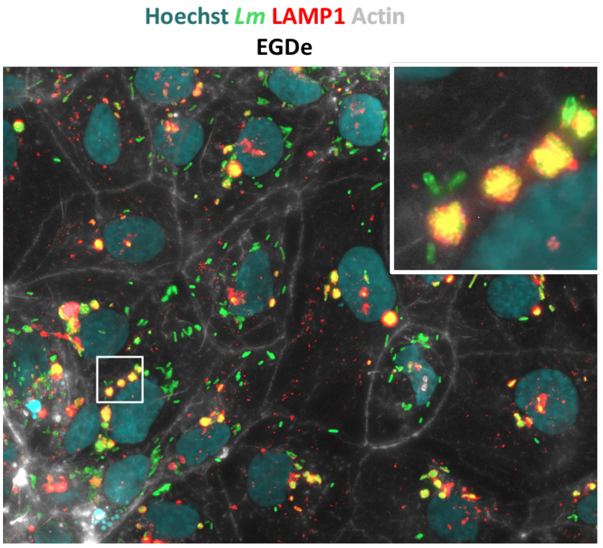

L76. Both InlA and InlB are internalin family proteins. However, internalin specifically indicates InlA, InlB is not called internalin B. Better to say "internalin proteins InlA and InlB" here.

Corrected.

Fig. 1D. From the text we know the images were from cells infected by Lm for 72 hours. It is better to mention the information in the figure and/or the legends to help the readers.

We have now included this information in the figure legend.

L212-213. I am not sure if the results support the conclusion that "InlA-Δ isolates are a better source to find genes involved in intracellular infection." This could be due to other factors in the genetic background of CC9 since all the identified strain are all of CC9 but none from CC121.

Our initial statement was based on the fact that in the first screening, variant V1 was isolated from a CC121 background. However, as the reviewer correctly pointed, in our second screening no additional CC121 variants

were identified. In light of this, we have revised the text to tone down our conclusion. The manuscript now states: “This finding suggests that either InlA-Δ isolates constitute a suitable source for identifying genes involved in intracellular infection, or that the CC9 genetic background, to which all identified variants belong, harbors specific factors that influence bacterial persistence” (Lines 226–229). We have also updated the discussion accordingly (Lines 426-430).

L214 and fig 2C. As shown in Fig 1D and 2C, Lm can be LAMP1 associated and polymerize actin in the cytosol in one single infected cell. Can the authors provide the proportion of LAMP1-colocalized Lm cells so that the contribution of lysosomal Lm can be highlighted? Location of the inset images in the larger field images shall be indicated.

As requested, we have included the quantification of the proportion of LAMP1-positive bacteria in Fig 1D. We also attempted to perform a similar analysis for Fig 2C; however, this was not feasible under the experimental conditions used: infections with V2, V3, and V4 exhibit very low cytotoxicity, resulting in highly confluent cell layers with dense and continuous LAMP1 staining. Although LisCVs could be readily identified in infected cells, the compact LAMP1 signal prevented reliable image segmentation, precluding accurate automated quantification of global LAMP1–bacteria colocalization.

To circumvent this technical limitation, we quantified the proportion of actin-positive bacteria. As previously reported, actin-positive bacteria correspond to cytosolic bacteria that escaped lysosomal targeting (Kortebi et al *PloS Pathogens*, 2017). This complementary readout confirmed that, in screening 2, V2 displayed reduced actin association (therefore more LAMP1 association), whereas V3 and V4 exhibited the opposite phenotype, with increased actin labeling (less LAMP1 association). We have included these quantifications in Figure 2C and explained this in the text (please see lines 233-242)

L217 and L218. I assume the authors wanted to highlighted the identification of isolates in nature with altered intracellular persistence. However, we already know that non-hemolytic isolates deficient in LLO function are all defective in intracellular infection. The claim shall be refined.

We are not sure we understand the reviewer’s comment. We agree that non-haemolytic isolates lacking a functional LLO are already known to be defective for intracellular infection. By restricting our screen to the InlA-Δ CC, our goal was to identify mutants defective in intracellular replication—indeed, this would include classical examples such as LLO-deficient strains, as the reviewer notes. However, after establishing this selection framework, we were specifically interested in isolating mutants that were affected in long-term persistence within LisCVs. We have rephrased the manuscript to make more clear that the screen was designed initially to capture mutants broadly defective in intracellular growth, but ultimately aimed to identify those selectively altered in the maintenance of persistent vacuolar stages (lines 214-218).

Fig. 3ABC. LisCV size of actA mutant has not been quantified. Would be better to have it here.

We have included the *actA* deletion mutant in all three panels. V2 and *actA* mutant display highly similar phenotypes, both in terms of LisCV size and the number of bacteria contained within individual vacuoles. Analysis of infection kinetics revealed a stronger phenotype for the *actA* mutant, characterized by a more pronounced reduction in intracellular CFUs over time. This difference is consistent with the complete loss of *actA* in the deletion mutant, whereas V2 exhibits attenuated *actA* expression.

L233-234 and S3 fig. The data do not support the description. Instead, the result shows reduced infection, which can be due to reduced dissemination or initial invasion. Time-lapse recording is needed to confirm that V2 remain confined to the initially infected cells.

We agree with the reviewer that our statement is not fully supported by a direct experiment. However, we would like to highlight that, as shown in Fig. 3C, the V2 strain has an invasion capacity comparable to that of EGDe-*ΔinlA* mutant. Together with the data presented in Fig. 3D–G, our results indicate that the observed phenotype is more likely due to reduced actin polymerization and impaired cell-to-cell spreading at 6 hours post-infection. We have revised the manuscript accordingly to clarify this point, now stating: “Consistently, microscopy observations of infected cells revealed that V2 was strongly impaired in cell-to-cell spread at the very early stage of infection” (lines 259–260). Additionally, we have updated the figure legend to reflect these changes (lines 1101).

L259-262. Any evidence for PrfA activation defect in V2? Is expression of any other genes in PrfA regulon, in addition to actA, affected in V2?

The regulation of the PrfA regulon by GshF has been extensively characterized in previous studies (Reniere *et al* *Nature* 2015; Reniere *et al* *PloS Pathogens* 2016; Portman *et al* *mSphere* 2017). Given this well-established relationship, we believe that additional experiments to assess *prfA* activation or the expression of other PrfA regulon genes in the V2 strain are not strictly necessary for the scope of our current work.

L269. Less for uncountable nouns. Use “fewer” here.

Thanks. Corrected.

L336-337. Including the growth curve in the supporting information is helpful

We have added the growth curves for V3, V4 and the *folP* L188S mutant, as requested. Please refer to the new Fig S6.

S1A and S1B movies. Precise time recording information showing time axis in the video is needed.

We have included this information in both movies.

L508-510. Different MOI was used for CC9, CC121 and the reference strains for screening. Was this the case for other experiments? Better to mention this in the result and figure legends for clarification.

We have adapted MOI for each experiment in order to have similar entry rate. This information is now added to all figure legends.

Reviewer #2: -line 64, the paragraph seems to end after one sentence… typo?

We thank the reviewer for bringing this typo to our attention. Corrected.

-Fig1D. What timepoint is shown in the IF images (should be stated in figure legend).

We have added this information in the figure legend (72h).

-line 179, “must be due to the acquisition of one or more mutations”. Recommend changing to “might be due”.

Corrected, thanks.

-line 370 “Some bacteria can even adopt a dual life style”… is there a good example the authors can discuss? Importantly, what is known about the genetic and metabolic factors that govern which lifestyle is utilized?

We thank the reviewer for this comment. There are actually many good examples! We have added a couple of references that review the different niches of intracellular pathogens as well as the genetic features that regulate their lifestyle (Petit and Lebreton, *Trends Microbiol* 2022; Gutierrez and Enninga, *Current Opinion Microbiol* 2022).
